# Differential aquaporin 4 expression during edema build-up and resolution phases of brain inflammation

**DOI:** 10.1186/1742-2094-8-143

**Published:** 2011-10-19

**Authors:** Thomas Tourdias, Nobuyuki Mori, Iulus Dragonu, Nadège Cassagno, Claudine Boiziau, Justine Aussudre, Bruno Brochet, Chrit Moonen, Klaus G Petry, Vincent Dousset

**Affiliations:** 1 INSERM U.1049 Neuroinflammation, Imagerie et Thérapie de la Sclérose en Plaques, F-33076 Bordeaux, France; 2 CHU de Bordeaux, Service de Neuroimagerie Diagnostique et Thérapeutique, F-33076 Bordeaux, France; 3 CNRS, UMR 5231 Laboratoire d'Imagerie Moléculaire et Fonctionnelle, F-33076 Bordeaux, France

**Keywords:** Aquaporin 4, Blood brain barrier, Brain edema, Inflammation, Magnetic resonance imaging

## Abstract

**Background:**

Vasogenic edema dynamically accumulates in many brain disorders associated with brain inflammation, with the critical step of edema exacerbation feared in patient care. Water entrance through blood-brain barrier (BBB) opening is thought to have a role in edema formation. Nevertheless, the mechanisms of edema resolution remain poorly understood. Because the water channel aquaporin 4 (AQP4) provides an important route for vasogenic edema resolution, we studied the time course of AQP4 expression to better understand its potential effect in countering the exacerbation of vasogenic edema.

**Methods:**

Focal inflammation was induced in the rat brain by a lysolecithin injection and was evaluated at 1, 3, 7, 14 and 20 days using a combination of in vivo MRI with apparent diffusion coefficient (ADC) measurements used as a marker of water content, and molecular and histological approaches for the quantification of AQP4 expression. Markers of active inflammation (macrophages, BBB permeability, and interleukin-1β) and markers of scarring (gliosis) were also quantified.

**Results:**

This animal model of brain inflammation demonstrated two phases of edema development: an initial edema build-up phase during active inflammation that peaked after 3 days (ADC increase) was followed by an edema resolution phase that lasted from 7 to 20 days post injection (ADC decrease) and was accompanied by glial scar formation. A moderate upregulation in AQP4 was observed during the build-up phase, but a much stronger transcriptional and translational level of AQP4 expression was observed during the secondary edema resolution phase.

**Conclusions:**

We conclude that a time lag in AQP4 expression occurs such that the more significant upregulation was achieved only after a delay period. This change in AQP4 expression appears to act as an important determinant in the exacerbation of edema, considering that AQP4 expression is insufficient to counter the water influx during the build-up phase, while the second more pronounced but delayed upregulation is involved in the resolution phase. A better pathophysiological understanding of edema exacerbation, which is observed in many clinical situations, is crucial in pursuing new therapeutic strategies.

## Background

Brain vasogenic edema is of central importance in the pathophysiology of a wide range of brain disorders [1]. In many pathologies, vasogenic edema is a highly dynamic process with phases of significant water accumulation and subsequent reduction. This process is seen in infectious and inflammatory disorders such as encephalitis, with edema peaking during the active phase. Other examples include severe stroke [2] and brain trauma [3], which are accompanied by vasogenic edema peaking at about 72-96 hours after insult and the risk for a significant elevation of interstitial pressure, herniation and death. A better understanding of the pathophysiology of such exacerbation of edema is crucial in pursuing new therapeutic strategies.

Edema pathophysiology can be viewed as a balance between formation and resolution [4]. Most research on this topic has concentrated on edema fluid formation. It has been established that breakdown of the blood-brain barrier (BBB) to plasma proteins is the leading determinant of water accumulation within the extracellular space [5]. Numerous and frequently interdependent mechanisms can contribute to the loss of BBB integrity [2]. One important common determinant of increased paracellular permeability is brain inflammation. Because brain inflammation occurs in a phasic manner, water entrance secondary to inflammation is thought to contribute to the ongoing clinical exacerbation that is observed following stroke, trauma or encephalitis [6]. In contrast, less is known about the mechanisms of edema fluid elimination. Edema fluid can be cleared into the cerebrospinal fluid (CSF) in the subarachnoid space or ventricles, or it can be cleared back into the blood [7]. All of these exit routes strongly express the selective water channel transporter aquaporin 4 (AQP4) [8]. Experiments that were conducted on AQP4-null mice have shown that AQP4-dependent transmembrane movements into the CSF and blood are dominant mechanisms for clearing excess brain water in vasogenic edema [9-11]. Therefore, the regulation of AQP4 expression could be an important determinant of the overall water content based on its involvement in the resolution of edema. There have been several reports of altered AQP4 expression in astrocytes in cases of brain edema [8]. The severity of the disease producing interstitial edema was associated with the upregulation of AQP4, which could potentially be a protective mechanism for countering edema accumulation [12]. Nevertheless, a precise temporal course of this AQP4 upregulation during the build-up and resolution phases in the dynamic evolution of vasogenic edema *in vivo *is still lacking.

This study sought to determine the time course of AQP4 expression in direct relation to interstitial water content. More specifically, we questioned whether AQP4 was differentially modulated during edema formation and resolution. We chose an inflammatory model because brain inflammation can be considered as a common determinant of vasogenic edema formation and exacerbation in many disorders, and we used magnetic resonance imaging (MRI) to assess *in vivo *the water content that was directly related to AQP4 expression. We found the more significant transcriptional and translational upregulation of AQP4 only during the edema resolution phase, with AQP4 being potentially insufficient to counter the excess water accumulation that occurs during the initial edema build-up phase.

## Methods

### Animal model of inflammatory vasogenic brain edema

All of the experiments were performed in accordance with the European Union (86/609/EEC) and French National Committee (87/848) recommendations (animal experimentation permission: France 33/00055). Male Wistar rats weighing 250-300 g were maintained under standard laboratory conditions with a 12-hour light/dark cycle. Food and water were available *ad libitum*.

A stereotaxic injection of L-α-lysophosphatidylcholine (LPC) stearoyl (Sigma, France) was used to create a focal demyelination that was associated with an inflammatory reaction around the site of the injection with a breakdown of the BBB [13]. Rats were anesthetized with an intraperitoneal injection of pentobarbital (1 ml/kg of a 55 mg/ml solution *i.p*.) and were immobilized in a stereotaxic frame (David Kopf, California). Injection coordinates were measured from the bregma to target the right internal capsule and were 1.9 mm posterior, 3.5 mm lateral and 6.2 mm deep. A 33-gauge needle attached to a Hamilton syringe that was mounted on a stereotaxic micromanipulator was used to inject LPC through a small hole drilled into the skull. An injection of 20 μl of 2% LPC (previously diluted with sterile serum and 0.01 M guanidine to increase its solubility and diffusion) was conducted slowly over a 60-minute period. Once the solution was infused, the cannula was slowly removed, and the incision was stitched. The day of injection was assigned as day 0.

Four groups of animals were studied at five time points following the LPC injection: 1, 3, 7, 14 and 20 days post-injection (dpi). The first group of animals (n = 25) underwent MRI and was sacrificed at the predefined time points (n = 3 to 6 per group) with an intracardiac perfusion of 4% paraformaldehyde (PFA) in 0.1 M phosphate-buffered saline (PBS) to assess MRI-co-registered histological analyses. A second group was injected with NaCl 0.9% and guanidine 0.01 M but without LPC (sham animals, n = 10) and followed by MRI prior to histological analyses (n = 2 rats per time point (t), with one additional MRI scan at the previous time point (t-1) per rat, *i.e*. n = 4 MR scans per time point). The third group of animals (n = 24) was sacrificed prior to (basal expression) and at the same time points after LPC injection (n = 3 to 5 per group) to collect fresh brains for the measurements of AQP4 expression by reverse transcription quantitative real-time PCR (RT-qPCR) and western blot experiments. The last group of animals (n = 15 with n = 3 per group) was used to study the patency of the BBB by the quantification of Evans blue extravasation according to a previously published method [9].

### MR Imaging

#### MRI protocol

Animals were investigated with MRI at 1, 3, 7, 14 or 20 dpi (assigned as time (t)) and then immediately sacrificed. The same animals were also investigated with MRI at the prior time point (t-1) to allow for the comparison of the data obtained at a single time points from two different series of rats and to consequently ensure the required level of reproducibility in the model for extrapolating longitudinal curves. Five animals that were sacrificed at later time points (14 and 20 dpi) were further scanned with MRI three times to longitudinally illustrate the time course of edema and to confirm the cross-sectional data (total MRI, n = 49). Images were obtained using a 1.5-Tesla magnet (Philips Medical System, Best, Netherlands) equipped with high-performance gradients, using a superficial coil (23-mm diameter). Anesthesia was induced with pentobarbital (1 ml/kg of a 55 mg/ml solution *i.p*.), and coronal sections were obtained using T2- and diffusion-weighted imaging (DWI).

T2-weighted images (T2WI) were obtained using the following parameters: fast spin-echo sequence, 10 slices, 1.5-mm thick, FOV = 5 × 1.75 cm^2^, reconstructed matrix = 256^2^, TR/TE/α = 1290/115 ms/90°, TSE factor = 12, NEX = 22, duration = 6 min, 42 s.

DWI was performed with a multi-shot spin-echo Echo Planar Imaging sequence using the following parameters: 10 slices, 1.5-mm thick, FOV = 5 × 1.75 cm^2^, reconstructed matrix = 128^2^, TR/TE/α = 2068/43 ms/90°, EPI factor = 3, NEX = 2, duration = 8 min, 10 s. Gradients with two different b values (0 and 600 s/mm^2^) in the x, y and z axes were used. By averaging the images obtained for the three diffusion-weighted directions (b = 600 s/mm^2^), trace DWIs were generated for each section with the corresponding apparent diffusion coefficient (ADC) map.

#### MR image analysis

We used ADC, which reflects the Brownian motion of water molecules and indirectly water content, to monitor disease progression. Data processing was performed with ImageJ software (NIH freeware, http://rsb.info.nih.gov/ij/).

The lesion was assessed as high signal intensity on the T2WI. We first manually delineated the right internal capsule hypersignal on the T2WI. Within this delineation, the final lesion was automatically defined using a threshold > mean + 2 × SD as derived from the corresponding area in the unaffected hemisphere. This mask was propagated on ADC maps to measure the mean ADC lesion. As an LPC injection can create a central cavity (necrosis) at the injection site with inflammation developing at the periphery, an upper ADC threshold (1700 μm^2^/s) was used to eliminate these voxels. In a separate analysis, cavitation as assessed by the area of pixels with a fluid-like signal (ADC > 1700 μm^2^/s), was measured over time. All MRI data were then re-read with the corresponding histology to ensure a direct symmetry between the region of interest (ROI) for the ADC and the histological parameters and to address a direct MRI/histological comparison. The mean ADC was also measured in the symmetric contralateral hemisphere with the same threshold.

### Histology

Rats were sacrificed for histological examination immediately following the final MR exam. Brains were removed following PFA perfusion, post-fixed for 24 h in the same fixative and then a 5-mm block across the injection mark was cut (coronal sections, 30-μm thick) with a vibratome (Leica, Switzerland). The extent of the parenchyma alteration was evaluated using luxol fast blue Kluver Barrera coloration to detect myelin and nuclear cells. Immunostaining was performed against AQP4, ED1 and Iba1 (for macrophages and microglia), IgG (for serum protein accumulation secondary to BBB alteration) and GFAP (for astrocytes).

#### Immunostaining

For immunohistochemistry, we used affinity-purified mouse monoclonal antibodies for ED1 (Serotec, 1/100) and rabbit polyclonal antibodies for AQP4 and GFAP (Sigma, 1/100 and Dako, 1/1000, respectively). Immunostaining was conducted in PBS containing 0.1% Triton X-100 and 3% swine serum. Revelation was performed with diaminobenzidine (DAB; Vector Kit, Vector Laboratories, USA) and nickel. Floating sections were rinsed, mounted on slides, and cover-slipped with Eukit medium.

For immunofluorescence, double-labeling was performed using a mixture of two primary antibodies [(polyclonal anti-AQP4 1/100 and monoclonal anti-GFAP 1/1000) or (polyclonal anti-Iba1 (Wako, 1/1000) and monoclonal anti-ED1 1/1000)] overnight at 4°C followed by a mixture of two secondary antibodies (anti-rabbit coupled to CY3 (Sigma, 1/300) and anti-mouse coupled to Alexa 488 (Sigma, 1/2000 or 1/1000)) for 2 h at room temperature (RT). For IgG leakage staining within the brain parenchyma, sections were incubated for 2 h at RT with an Alexa-488-conjugated affinity-purified donkey anti-rat IgG antibody (Invitrogen, 1/500). Immunofluorescence sections were mounted and cover-slipped using the VectaShield mounting medium (Vector Laboratories). For all immunostaining experiments, the staining specificity was examined by omitting the primary antibody during the corresponding incubation.

#### Immunostaining analysis

For comparison, both MRI and histological sections were perpendicular to the flat skull position. AQP4 immunolabeling was evaluated on serial slices that corresponded to the MRI acquisitions (three to four slices) using ImageJ software at the same level as the MRI measurements. Double staining for AQP4 and GFAP was examined using confocal laser scanning microscopy (Leica DM2500 TCS SPE on a upright stand, Leica Microsystems, Germany) using the following objectives: HCX PL Fluotar 20X oil NA 0.7 and HCX Plan Apo CS 40X oil NA 1.25 and diodes laser (488 nm, 532 nm). AQP4 immunoreactivity was quantified in three different fields (345 μm^2^) that were positioned within the lesion excluding central cavitation, and symmetrically within the left hemisphere. The analysis was performed on 0.7 μm thick images (n = 8 z positions for each field), keeping a constant laser power and gain. AQP4 staining was thresholded to eliminate background signals, and the results are reported as the mean area of immunoreactivity. The results were further controlled using the ImageJ "mean gray" tool on raw images (non-treated images) and reported as a ratio using "mean gray" in the contralateral hemisphere. There was no change in AQP4 expression in the contralateral internal capsule of LPC rats (nor in the sham group), consistent with a previous focal infectious/inflammatory model of brain abscess that displayed AQP4 modification only in a ring surrounding the lesion [9]. Thus, ratio analysis using the contralateral hemisphere as an internal reference was appropriate to minimize the confounding effects of possible differences in fixation efficiency from one animal to another. The same procedure was used for GFAP and ED1 labeling by looking at the mean immunoreactivity of the slices revealed by DAB within lesioned and contralateral fields. ED1/Iba1 and IgG immunofluorescence preparations were examined by epifluorescence microscopy (Nikon) using the 488-nm (Alexa) and 568-nm (CY3) channels. For IgG staining, full sections were digitized with a CCD camera coupled to the microscope to measure the area of BBB leakage on six slices covering the entire lesion.

### RT-qPCR experiments

We quantified AQP4 mRNA along with interleukin-1β (IL1β) as a marker of active inflammation and GFAP (astrocytes) as a marker of glial scarring following the MIQE guidelines [14]. Brains were freshly extracted following transcardiac PBS perfusion. A 3-mm-thick coronal section (approximately -0.4 mm to -3.4 mm from the bregma) was dissected around the injection mark. Macro-dissection of the tissue bordering the internal capsule was performed with a 3-mm-core unipunch in the lesioned and contralateral side. Tissue samples (mean weight 40 to 50 mg) were immediately snap-frozen in liquid nitrogen vapor, stored at -80°C, and RNA was isolated using Trizol reagent (Sigma) according to the manufacturer's protocol and re-suspended in 20 μl RNase free water. The RNA concentration was calculated by spectrophotometric analysis (NanoDrop; Thermo Scientific). The quality of extraction was assessed by the A260/A280 and A260/A230 ratios, which were always ≥1.8, and by electrophoresis on a 1.5% agarose gel. The absence of significant DNA contamination was assessed with a no-reverse transcription assay.

50 ng of RNA was reverse-transcribed to cDNA using Sensiscript^® ^reverse transcriptase (Qiagen, France) for AQP4 and GFAP and 2 μg of RNA was reverse-transcribed using Omniscript^® ^(Qiagen, France) for IL1β. Reverse transcription was carried out in a total volume of 20 μl containing 2 μl oligo dT, 5 μM in 2 μl of 5 mM dNTP and 1 μl reverse transcriptase in 2 μl 10x buffer diluted in distilled water. The reaction was allowed to proceed at 37°C for one hour and was terminated by heating to 95°C for three minutes.

The primer sequences for the PCR reactions are shown in the Table 1. Samples from each rat were run in triplicate and quantified using a Bio-Rad iCycler real-time PCR system. Each sample consisted of 5 μl cDNA diluted 1/20, 12.5 μl Mesa Green qPCR buffer (Taq DNA polymerase, reactive buffer, dNTP mix, 4 mM Mg Cl_2 _and SYBR Green I from Eurogentec, France), 0.25 μl each of forward and reverse primer (10 μM working dilution) in double distilled water to a final volume of 25 μl. The amplification protocol consisted of one cycle at 95°C for 3 min, followed by 40 cycles at 95°C for 10 sec, 65°C for 1 min, and finished by 55°C for 30 sec. Specificity previously assessed *in silico *(BLAST software) was confirmed by electrophoresis and the observation of a single peak after the Melt^® ^procedure. Quantification cycles (Cq) were determined with the Bio-Rad software and the Cq of the no-template control was always >40. The results were analyzed using the comparative Cq method for the experimental gene of interest normalized against the reference gene GAPDH [15], which showed an invariant expression under the experimental conditions described (standard deviation of GAPDH Cq <0.5).

**Table 1:** Primer sequences used in RT-qPCR

Gene	Accession number	Primer sequences from 5' to 3'	Location of amplicon	Amplicon length	Efficiency
AQP4	Isoform 1: NM_012825.3	Sens: TTGGACCAATCATAGGCGC	770 to 788 Isoform 1	213 pb	98.2%
			778 to 796 Isoform 2		
	Isoform 2: NM_001142366.1	Revs: GGTCAATGTCGATCACATGC	963 to 982 Isoform 1		
			971 to 990 Isoform 2		
GFAP	NM_017009.2	Sens: GCGGCTCTGAGAGAGATTCG	692 to 711	90 pb	102.0%
		Revs: TGCAAACTTGGACCGATACCA	761 to 781		
IL1β	NM_031512.2	Sens: AATGACCTGTTCTTTGAGGCTGAC	111 to 134	115 pb	91.2%
		Revs: CGAGATGCTGCTGTGAGATTTGAAG	201 to 225		
GAPDH	NM_017008.3	Sens: TGCTGGTGCTGAGTATGTCGTG	337 to 358	101 pb	89.5%
		Revs: CGGAGATGATGACCCTTTTGG	417 to 437		

### Western blot

Proteins were extracted from the phenol-chloroform phase of the Trizol procedure and homogenized in 1% SDS. Protein quantification was performed using the Micron BCA™ protein assay reagent kit (Pierce). Protein samples (7 μg) were separated by an SDS PAGE gel (10%) at 100 V for 80 min on a minigel system (Bio-Rad). Proteins were then transferred from the gel to a PVDF membrane (Immobilon-P transfer membrane, Millipore) at 100 V for 80 min. Non-specific sites on the membrane were blocked one hour at RT in a milk solution diluted in TBS/Tween. Primary AQP4 antibodies (1/500) and rabbit anti-actin antibodies (Sigma, 1/4000) were applied to the membrane for one hour at RT, followed by four rinses with TBS/Tween and a one hour incubation with 1/16000 dilution of peroxidase-labeled goat anti-rabbit at RT. Immuno-reactive bands were visualized using the ECL detection system (Pierce), and the intensities were determined by densitometry at bands of approximately 31 KDa for AQP4. Lane loading differences for each sample were controlled for by the normalization to the corresponding actin signal.

### Evans blue extravasation

At the defined time points (1, 3, 7, 14 and 20 dpi; n = 3 per time point), 40 mg/kg of Evans blue dye (solution 20 mg/ml) was injected via the tail vein. After 2 h, the brains were extracted following a PBS perfusion that was used to eliminate any circulating Evans blue. The tissue was homogenized in 700 μl of N;N-dimethyl formamide (Merck). The homogenate was centrifuged at 16000 g at 4°C for 20 min, and the supernatant was plotted in triplicate in a 96-well flat-bottom plate. The amount of Evans blue was measured spectrophotometrically at the 620 nm wavelength and determined by a comparison with readings obtained from standard solutions Data was expressed as μg Evans blue per g brain tissue. Prior to brain homogenization, representative qualitative images of Evans blue extravasation from PBS perfused brains were taken using a digital camera.

### Statistical analysis

Analyses were performed using R software (version 2.11.1). All data are presented as the mean ± SD or as medians and quartiles (Q1-Q3). For the edema time course, we first compared ADC in the injured hemisphere at 1 dpi to corresponding values taken in the contralateral hemisphere using the Wilcoxon test. We then compared ADC in the injured hemisphere from one point with another (1, 3, 7, 14 and 20 dpi) to explore the time course using a one-way analysis of variance (ANOVA) with the Bonferroni post-hoc test. From these analyses, we defined an edema build-up phase (significant ADC increase) and a resolution phase (significant ADC decrease). AQP4 and other markers (IgG, IL1β, GFAP, ED1, Evans blue amount, cavitation pixels with ADC > 1700 μm^2^/s) were studied over time by applying the same procedure. These molecular markers were compared between the MRI-defined build-up and resolution phases using the Mann-Whitney test. P values <0.05 were considered significant.

## Results

### Time course of LPC-induced lesions

**Figure 1:**
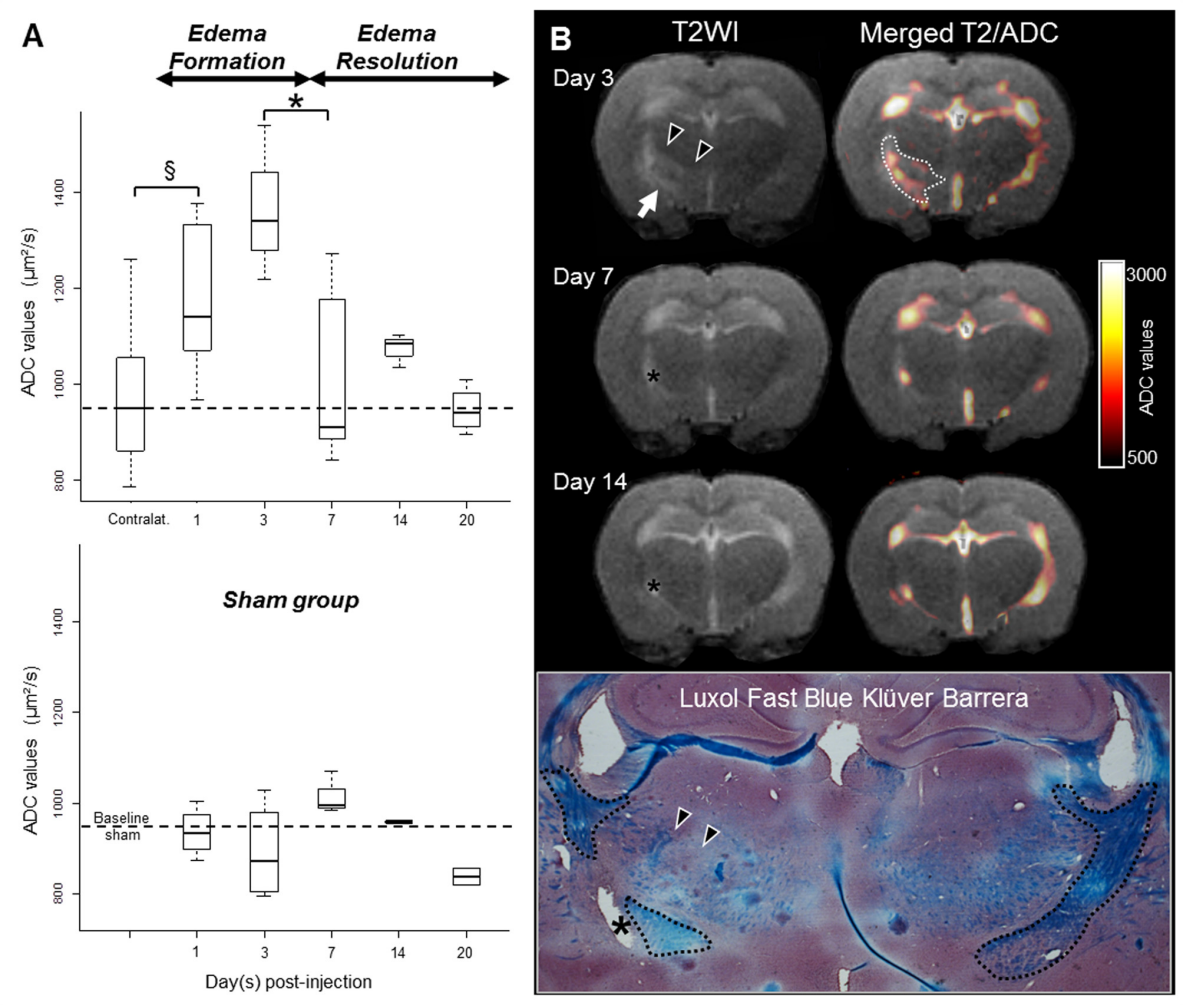
**Time course of LPC-induced edema as assessed by ADC measurements**. (**A**) Quantification of ADC values (median, Q1-Q3) revealed a biphasic evolution (ANOVA) with a first phase characterized by a rapid increase in water content (§, p = 0.006, Wilcoxon test) peaking at 3 dpi, corresponding to the active phase of inflammation. The second phase was characterized by water resolution (*, p = 0.015, ANOVA), with ADC values that returned to baseline during the formation of a glial scar. ADC values of sham rats were stable over time and were not different from those measured in the contralateral side of LPC rats. The dotted line is the median value over the 5 time points for the sham group. (**B**) Representative illustration of the time course with T2WI (left panel) and merged T2/ADC maps (right panel) of the same animal taken at three different time points (3, 7 and 14 dpi) with corresponding histology at 14 dpi (Luxol Fast Blue coloration). A large area of edema with high ADC values was seen at 3 dpi along the right internal capsule (arrow) and spread through the extramedullary lamina and medial lemniscus tracts toward the midline (arrowheads). The majority of the edema was resolved by 7 and 14 dpi, with a slight cavitation at the site of injection (*) with cerebrospinal-fluid-like ADC values. Histological evaluation of the lesion at 14dpi confirmed the small cavitation (*) and showed large demyelination of the white matter tracts in which edema was initially observed. The myelin fibers of the internal capsule, stained in blue, were outlined (dotted lines) and a loss of myelin was seen in the internal capsule and also in the other white matter tracts (arrowheads).

In the sham treated group, ADC values were stable over time. Similarly, the MRI evaluation within the non-injected left internal capsule of LPC rats showed no T2 abnormalities and stable ADC values that were not different from those measured in the sham group (median ADC = 951.2 μm^2^/s for sham vs. 950.8 μm^2^/s for contralateral LPC; p = 0.54; Figure 1). Together, these data validate the contralateral side of LPC rats as an intra-individual control for each animal.

Within the right (injured) hemisphere of LPC rats, ADC values varied over time, and we identified two distinct phases: *(i) *an initial edema build-up phase and *(ii) *a later resolution phase. At the earlier time points (1 and 3 dpi), large areas of T2 signal increase were observed spreading within the internal capsule and also within other white matter tracts, such as the medial lemniscus and extramedullary lamina tracts toward the midline (Figure 1). At later time points (7, 14 and 20 dpi), the T2 hypersignal decreased and, occasionally showed a persistent cavitation area at the site of injection (Figure 1). Such cavitations (pixel with ADC value > 1700 μm^2^/s) were small and were significantly increased only at 20 dpi (mean area = 4.28 mm^2^, p = 0.005). Quantitative analysis of the edema time course with DWI confirmed a significant variation in ADC over time (ANOVA, F = 5.21, Df = 4, p = 0.005), with a significant increase as early as 1 dpi (p = 0.006), a peak at 3 dpi and a secondary decrease between 3 and 7 dpi (p = 0.015). The ADC values at 7, 14 and 20 dpi returned to baseline and were not statistically different from those of the contralateral side (p = 0.34, Figure 1).

The ADC time course described above was derived from cross-sectional and independent data, proceeding from the MR scans conducted just before sacrifice (n = 25). By introducing the repetitive MR scans that were performed before sacrifice (two to three scans per animal except for 1 dpi, total = 49) and by evaluating the longitudinal data for each animal (Figure 1), the time course of edema build-up and resolution phases was confirmed.

### Build-up and resolution phase characteristics

**Figure 2:**
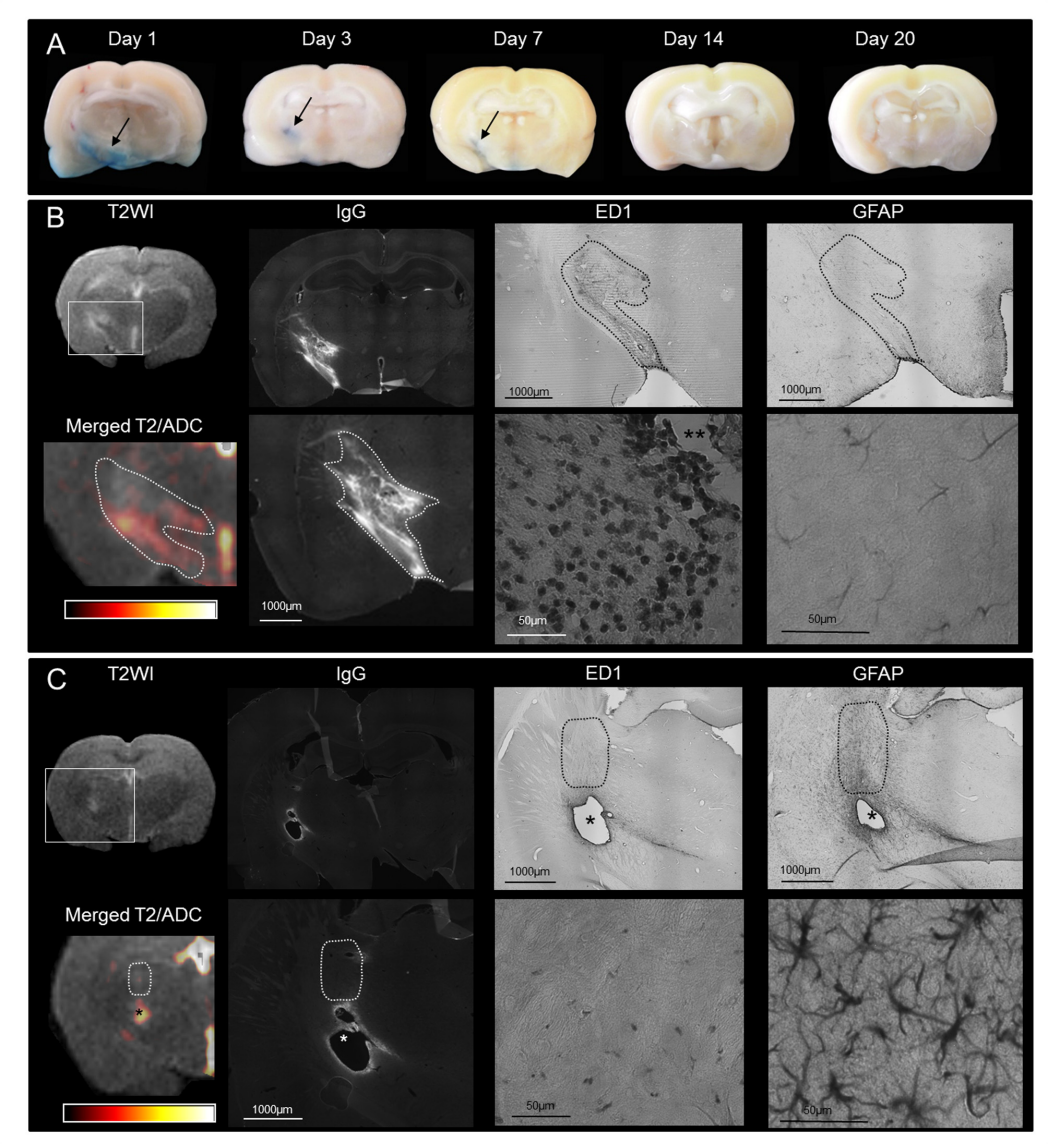
**Edema build-up and resolution phase characteristics**. (**A**) Representative samples of Evans blue extravasation from rats sacrificed at 1, 3, 7, 14 and 20 dpi. Widespread leakage at 1 dpi (arrow) progressively decreased with a restriction to the lesion site (3 and 7 dpi, arrows) followed by a complete restoration of the BBB integrity at the later time points (14 and 20 dpi). (**B**) and (**C**) are representative illustrations of MRI and histological features for rats explored at 1 dpi (**B**) and 20 dpi (**C**). During the edema formation phase (1 dpi, **B**), the T2 signal increased along the internal capsule up to the midline with high ADC values (similar pattern as in Figure 1, day 3). The corresponding histology showed important BBB permeability (IgG) and massive infiltration of ED1 + cells around vessels (**) in MRI-defined edematous areas (dotted lines) while astrocytes were faintly stained (GFAP). During the edema resolution phase (20 dpi, **C**), T2 and ADC signals were mostly normalized, with the only persistence of a small cavitation at the site of injection due to necrosis (*, similar pattern as in Figure 1, day 14). The corresponding histology showed a large area with hypertrophic and entangled astrocytes i.e., gliosis (GFAP) around the point of injection (dotted lines) while BBB leakage (IgG) had mostly resolved with much lower presence of ED1+ cells.

**Figure 3:**
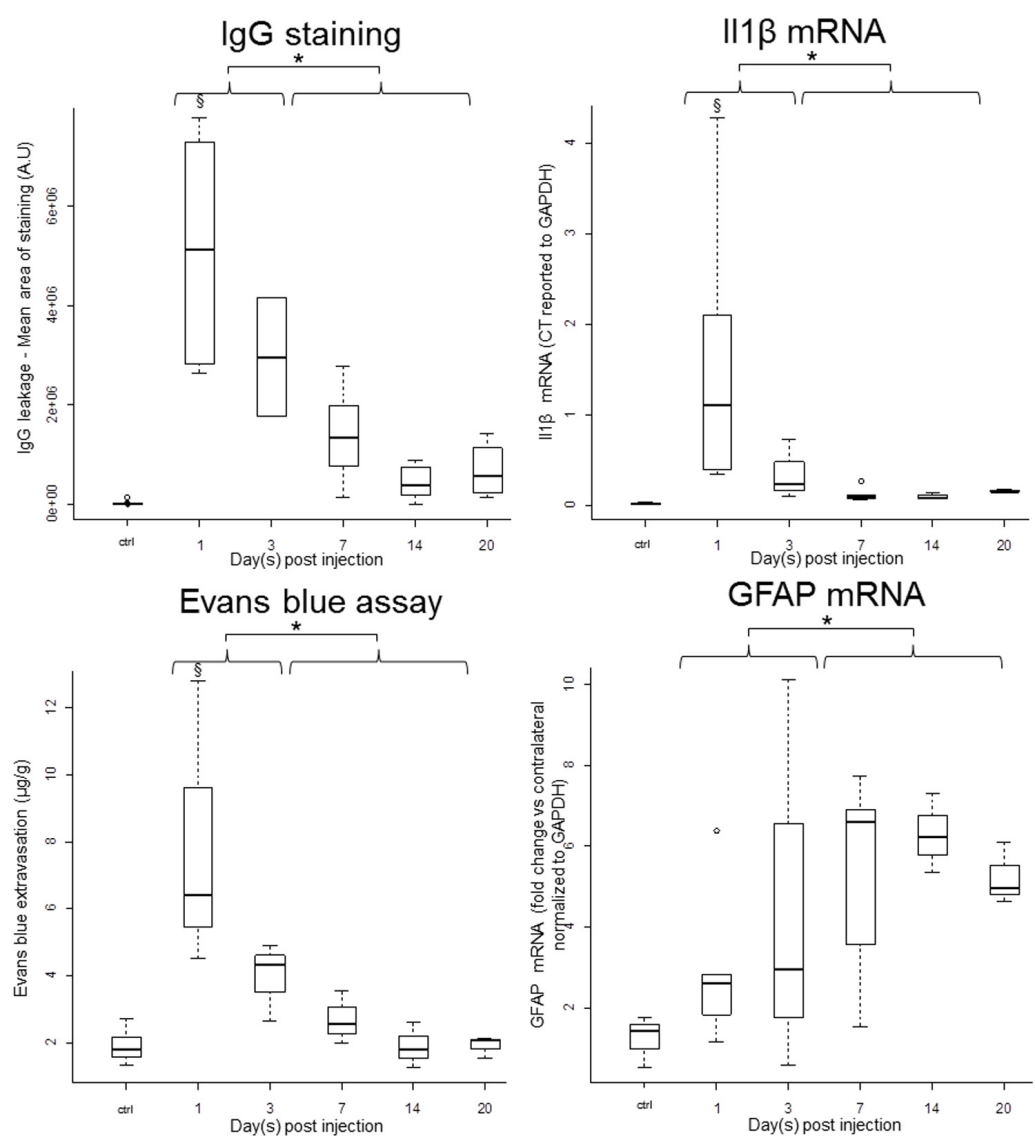
**Quantitative features of edema build-up and resolution phases**. Markers of BBB permeability (immunostaining of endogenous IgG extravasation and Evans Blue leakage) and pro-inflammatory cytokine (IL1β mRNA quantification) were found as early as 1 dpi (§, p < 0.05, Wilcoxon test) and were significantly increased during the build-up phase of the model compared to the resolution phase (*, p < 0.001, Mann Whitney). The resolution phase (7 to 20 dpi) was characterized by the formation of a glial scar with a significant increase of GFAP (mRNA quantification *, p < 0.05, Mann Whitney).

**Figure 4:**
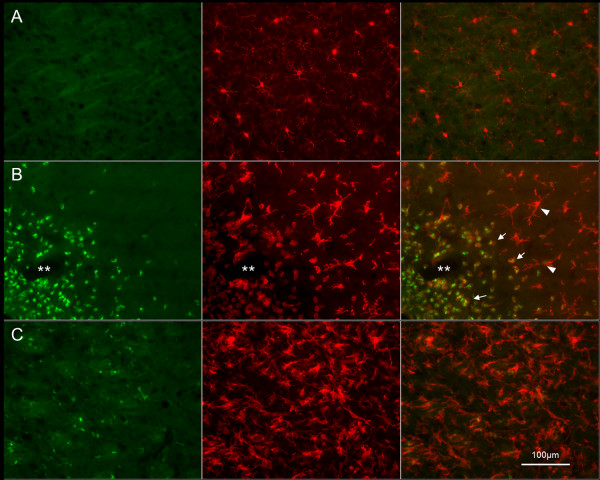
**Inflammatory cell subtypes**. Double labeling of ED1 (Alexa 488, green) and Iba1 (CY3, red) in the contralateral brain (**A**) and at the lesion site at 1 dpi (**B**) and 14 dpi (**C**). On the contralateral side (**A**), only resting microglia were stained with ramified thin processes and weak Iba1 immunoreactivity. During the edema formation phase (1 dpi, **B**), many round cells with both ED1 and Iba1 immunopositivity (arrows) were found around vessels (**) and were thought to be infiltrating macrophages, while some could also represent amoeboid microglia with a fully activated profile. At the periphery of the lesion, some activated microglia Iba + but ED1 - could also be observed (arrowheads). During the edema resolution phase (14 dpi, **C**), most cells were Iba1 + but ED1 - and showed highly branched processes corresponding to activated microglia.

During the edema build-up phase (1 and 3 dpi), inflammatory marker levels were significantly increased compared to the second resolution phase (Figures 2 and 3). In the areas that displayed water accumulation according to ADC maps, the Evans blue assay showed a significant BBB alteration leading to serum protein extravasation (IgG) as early as 1 dpi (p = 0.01 for Evans blue and p = 0.03 for IgG). The number of ED1+ cells progressively increased during the build-up phase. At this early phase, most ED1+ cells were round shaped and were often observed around blood vessels positively labeled for Iba1 (Figure 4). Based on their morphology and location, the majority of these cells were thought to be blood born macrophages, although some could also represent fully-activated microglia with an amoeboid shape. The pro-inflammatory cytokine IL1β mRNA was significantly increased as early as 1 dpi (p = 0.008) while the expression of GFAP was moderate.

During the edema resolution phase (7, 14 and 20 dpi), the levels of markers for scarring were significantly increased compared to during the build-up phase (Figures 2 and 3). BBB permeability progressively resolved with a significant disappearance of serum protein (p < 0.0001). The number of ED1 + cells significantly decreased (p < 0.0001), while many Iba 1+ cells with highly branched processes were detected; most were ED1- and corresponded to activated microglia with a profile suggestive of being more repair-oriented (Figure 4). The level of the pro-inflammatory cytokine IL1β was very low compared to during the build-up phase (p < 0.001). Glial scarring took place with an increase in GFAP mRNA expression (p = 0.01). Qualitative analysis from the histological sections demonstrated that astrocytes became hypertrophic and entangled and showed highly branched processes.

### Time course of AQP4 expression

In the sham group, no significant variation in AQP4 staining was observed over time, and no significant difference was found compared to the contralateral side of LPC rats.

**Figure 5:**
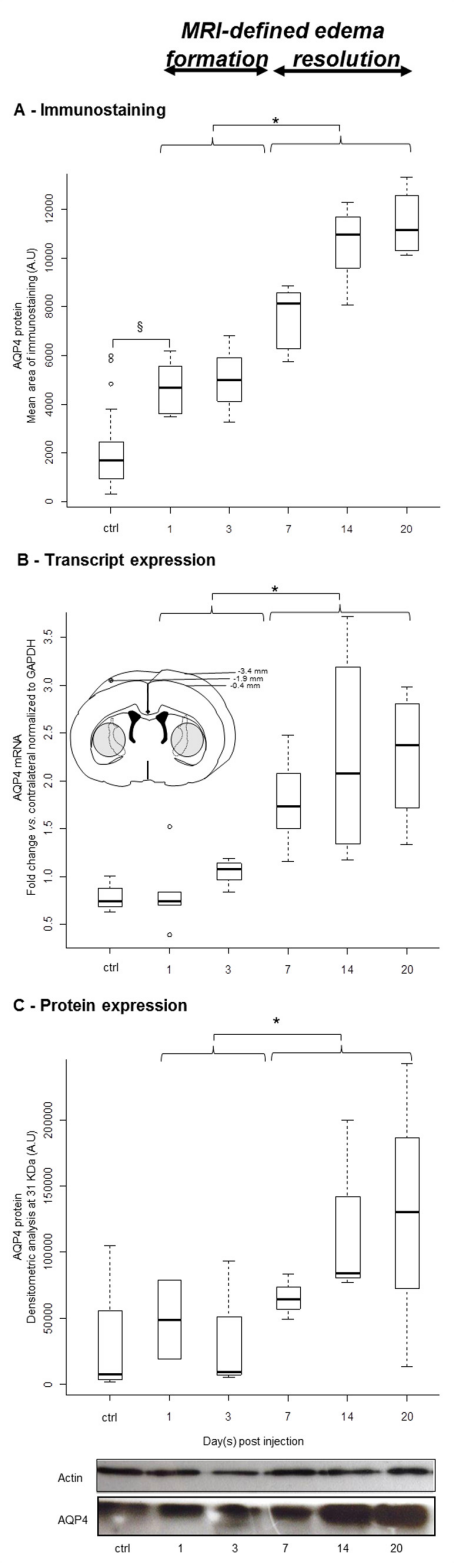
**Time course of AQP4 expression during edema formation and resolution**. (**A**) Histological evaluation depicted an initial upregulation of AQP4 as early as 1 dpi (§, p = 0.003, Wilcoxon test) that plateaued at 1 and 3 dpi. A significant increase in AQP4 expression was found during the MRI-defined edema resolution compared to the MRI-defined edema formation phase (*, p < 0.0001 Mann Whitney). RNA quantification (**B**) and protein quantification with western-blot (**C**) confirmed a much stronger increase in the expression of AQP4 during the MRI-defined edema resolution compared to the MRI-defined edema formation phase (*, p < 0.05 Mann Whitney). The inset in **(B) **shows the area of the tissue micro-dissection. A tissue block of 3 mm was cut around the injection site. Within the block, samples from the injured and contralateral sides were obtained using a 3-mm-core unipunch (right and left shaded circles). In **(C)**, a representative western blot shows the strong increase of AQP4 at 14 and 20 dpi, while actin, which was used to control loading variations, was stable.

In LPC operated rats, semi-quantitative histological analyses conducted in direct comparison and in the same ROIs as the MRI analyses revealed a moderate but significant increase in AQP4 at 1 dpi compared to the contralateral side (p = 0.003, Figure 5A). This initial upregulation was not observed using RT-qPCR or western blot methods conducted on the tissue lysates (Figure 5B and 5C). Then, quantitative analyses revealed a significant variation in AQP4 expression over time (ANOVA, p < 0.05), with higher levels of AQP4 expression observed during the edema resolution phase compared to the build-up phase as evaluated by immunostaining (p < 0.0001), RT-qPCR (p = 0.001) and western blotting (p = 0.034, Figure 5). Consistent results were observed using both histological analysis methods (staining area and mean gray ratio) and both RT-qPCR and western blot analysis methods (absolute values or ratios to the contralateral side).

**Figure 6:**
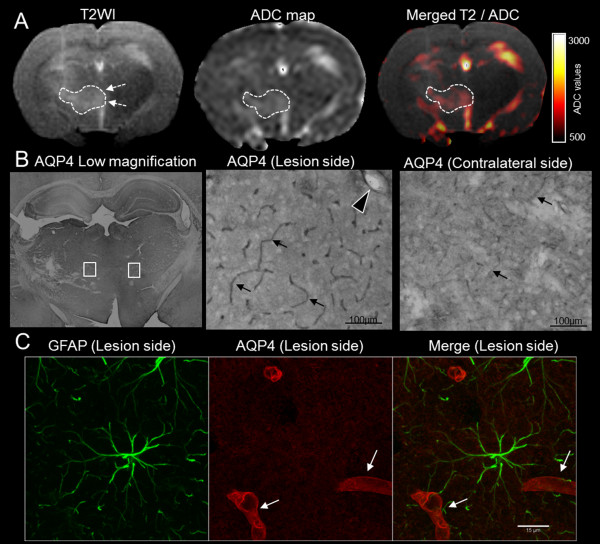
**MRI/histological correspondence during the build-up phase of edema**. A representative rat examined at 1 dpi is shown. (**A**) A large hypersignal area was seen on the T2WI (dotted line) with high ADC values (dotted line, ADC = 1377 μm^2^/s as opposed to 1039 μm^2^/s in the symmetric contralateral area), indicating increased water content. A slight midline shift resulted from the cerebral edema (dotted arrows on T2WI). (**B**) The corresponding histological sections (low magnification, with white boxes indicating higher magnification positions) showed an increase in AQP4 immunoreactivity in the MRI-defined edematous area, with staining located around the capillaries (arrows) and larger vessels (arrowhead) at the BBB level. AQP4 staining in the symmetric contralateral area is fainter around the capillaries (arrows). (**C**) Double labeling of GFAP (Alexa 488, green) and AQP4 (CY3, red), examined using confocal microscopy confirmed the perivascular location of AQP4 on astrocyte endfeet surrounding capillaries (arrows) without any AQP4 on the astrocyte body.

During the MRI-defined edema build-up phase (1 and 3 dpi), qualitative analysis revealed that AQP4 staining was highly concentrated within the astrocyte membrane domains that were facing blood vessels. This appeared as a co-localization of AQP4 and GFAP on perivascular astrocyte endfeet (Figure 6). Furthermore, comparison with the MRI showed a direct spatial correspondence, with increased AQP4 immunoreactivity found in areas where ADC was also increased (Figure 6).

**Figure 7:**
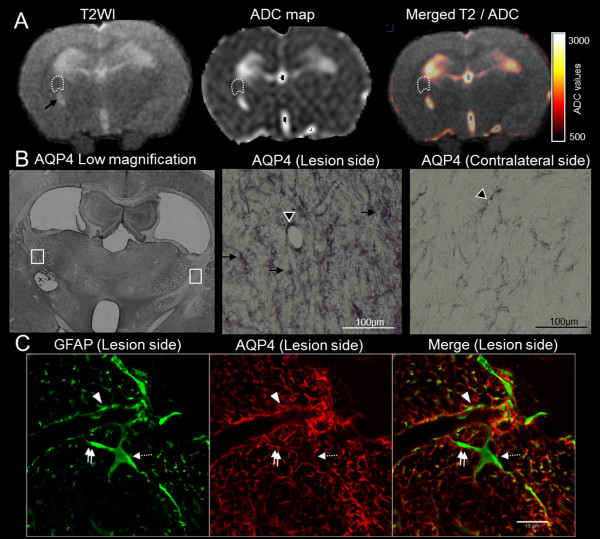
**MRI/histological correspondence during the resolution phase of edema**. A representative rat examined at 20 dpi is shown. (**A**) MRI showed a small cavitation at the site of the injection with a cerebrospinal-fluid-like signal on the T2WI (arrow) and ADC map, while no peripheral edema was anymore visible along the upper part of the internal capsule (dotted line, ADC = 889 μm^2^/s as opposed to 852 μm^2^/s in the symmetric contralateral area). (**B**) The corresponding histological sections (low magnification, with white boxes indicating higher magnification positions) showed a marked increase in AQP4 immunoreactivity, with staining located around the vessels (arrowhead) and with a fibrillary pattern corresponding to staining on the entire astrocyte membrane in a gliotic area (arrows). The staining in the symmetric contralateral area is more faint and only around capillaries. (**C**) Double labeling of GFAP (Alexa 488, green) and AQP4 (CY3, red), examined using confocal microscopy, confirmed AQP4 localization over the entire membrane of hypertrophic astrocytes expressing high levels of GFAP and not just around vessels (arrowhead). Double arrows show AQP4 staining along an astrocyte process and dotted arrows show AQP4 staining along an astrocyte cell body.

During the edema resolution phase (7, 14 and 20 dpi), the expression pattern was different from the first phase, with strong AQP4 expression throughout the entire membrane of astrocytes, rather than being confined to the domains facing blood vessels (Figure 7). Spatially, this expression pattern was observed on astrocytes that were located around the site of injection in areas where the ADC values had returned to normal (Figure 7).

## Discussion

**Figure 8:**
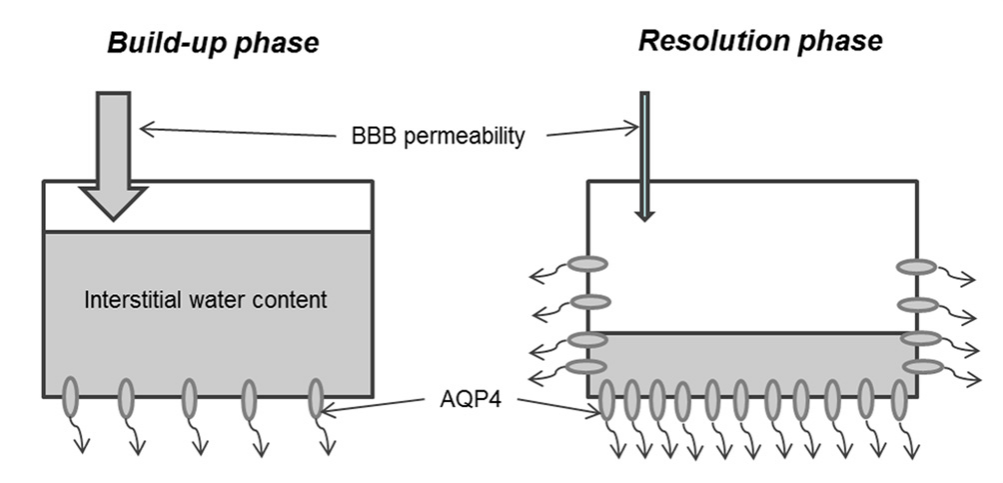
**Suggested model for interstitial edema pathophysiology**. The edema build-up phase results from high BBB permeability while AQP4 expression is not yet highly upregulated, resulting in insufficient routes for water elimination. After the time lag of AQP4 expression, edema resolution results from the conjunction of BBB restoration and subsequent significant AQP4 upregulation over the entire astrocyte membrane. Transition phases likely exist between the two extremes.

Exacerbation of vasogenic edema is feared in numerous clinical situations and is classically interpreted as the result of a modification of BBB permeability. Our study focused on AQP4 because of its role in the resolution of interstitial edema. We found that AQP4 expression was strongly up-regulated following an initial delay. This time lag in AQP4 upregulation could be a key determinant in the evolution of interstitial edema and could be associated with the worsening of a patient's condition. Following injury, a delay in efficient upregulation of AQP4 could result in the build-up phase of edema, as low AQP4 expression may be insufficient to counteract the opening of the BBB. On the other hand, the pronounced but delayed upregulation of AQP4 participates in the resolution phase of edema [11] (Figure 8).

Our knowledge of AQP4 involvement in brain edema can be approached in two different ways [8] regarding *(i) *the functions of AQP4 and *(ii) *its regulation of expression. *(i) *The functions of AQP4 in mammals have largely been determined by experiments using AQP4-null mice [10]. In models of cytotoxic edema, in which the BBB is intact, AQP4 deletion limits brain swelling by reducing the rate of edema fluid formation [16-19]. In contrast, in models of vasogenic edema, BBB breakdown is thought to be the major determinant of edema formation, independent of AQP4 [7]. In contrast to its beneficial role in cytotoxic edema, AQP4 deficiency generates more brain swelling in models of vasogenic edema, suggesting that water elimination occurs through transcellular, AQP4-dependent routes [9,11,20]. Each potential route of water exit (the BBB, glia limitans, and ependyma) strongly expresses AQP4 [21], explaining the impaired fluid clearance following vasogenic edema in cases of AQP4 deficiency. *(ii) *Second, several reports have examined the expression of AQP4 in different disorders that are associated with edema [22]. Discrepancy in the observation likely occurs due to the different models (cytotoxic, vasogenic, or even more complex situations combining cytotoxic and vasogenic edema) [8]. Furthermore, technical difficulties in water measurement and limited longitudinal data preclude a complete understanding of AQP4 regulation during build-up and resolution phases of edema. In a previous study using MRI as a sensor for edema, we reported an increase in AQP4 expression within the periventricular edema of hydrocephalic rats, with higher levels of AQP4 expression in more severe and chronic rats, findings that are consistent with our current results [12]. Nevertheless, in the hydrocephalus study, AQP4 expression was only associated with disease severity, but because the timing of the onset of hydrocephalus was unknown and because the hydrocephalus was not reversible (edema production continues over time), the time course of AQP4 expression during the build-up and resolution phases of edema could not be addressed. Furthermore, the edema of hydrocephalus had the same composition as cerebro-spinal fluid without serum protein, which did not allow an understanding of edema regulation associated with BBB alteration.

Edema exacerbation typically follows stroke [2], brain trauma [3] or encephalitis. Even if these incidents are very different in their initial stages, the secondary exacerbation of these pathologies is predominantly due to vasogenic edema [7]. Although the mechanisms for increasing BBB permeability and subsequent water entrance are complex and vary according to the exact pathophysiological situation, a secondary inflammatory reaction can be viewed as a shared determinant [23]. Consequently, we chose a purely vasogenic situation induced by inflammation as a clinically relevant model. LPC is a product of membrane degradation that acts as an inflammatory mediator [24] and is known to induce inflammatory reactions with demyelination [13]. We modified the classical protocol by using a higher injection dose. This protocol led to a more severe reaction, inducing the biphasic edema time course. The definite location and initiation of the lesion were additional key elements in the time course study. Furthermore, our modification to the model made it suitable for MR experiments and ADC measurement because larger lesions diminish the risks involved in partial volume averaging. MR *per se *offers several advantages regarding edema exploration. First, MR measurements such as ADC are quantitative, reproducible and highly validated, with an acceleration of diffusion (ADC increase) occurring when the overall water content is increased [25]. Second, because of the repetitive explorations of the same animal at different time points, we could ensure the reproducibility of the model and longitudinal information. Third, the MRI provided regional edema measurements that are impossible to attain with widely used methods such as the "wet and dry" weight technique [26]. The MRI approach allowed us to compare the water content with immunohistochemistry data in the same animal, including regional information, which is not possible with the wet/dry weight ratio method. Finally, as a non-invasive method used for patients, it affords a direct parallel to human disorders in translational research. Utilizing these properties, we found a direct spatial correspondence between edema as assessed by ADC and histological AQP4 expression modification.

In more detail, our data demonstrate a biphasic expression pattern of AQP4 that directly reflects the biphasic course of the edematous model. During edema build-up, a minor upregulation of AQP4 was seen only histologically with a perivascular location in MRI-defined edematous areas. RT-qPCR and western blot analysis did not show this early upregulation that may arise from a micro-heterogeneity in AQP4 expression that is increased mainly in regions exhibiting water accumulation. Spatial heterogeneity is taken into account with the direct histological/MRI comparison but such resolution could be lost within a larger sample (e.g., lysate of a tissue block). Alternatively, such a minor only histologically detectible increase in AQP4 could be related to the translocation of AQP4 to the endfeet with no alteration in overall AQP4 abundance. Either way, during this early phase, BBB dysfunction (as assessed by Evans blue) allowed an important passage of plasma proteins (IgG) and water (rapid increase of ADC), while AQP4 expression could be regarded as insufficient to handle such opening of the BBB and subsequent water influx. Thus, in the early phase (up to 3 days), AQP4 upregulation might represent a protective but insufficient response to limit brain swelling. Previous studies using stroke [27,28] and brain injury models [29,30] have reported an initial AQP4 downregulation. The cytotoxic nature of edema at early times in these models could account for the differences with our pure vasogenic model. If initial AQP4 downregulation could protect against intracellular entrance at early times [17], it could also induce a delay in the secondary upregulation that is necessary to clear the second phase of vasogenic edema exacerbation, which is consistent with our results.

During the resolution phase, the edema decrease coincided with a second more significant upregulation of AQP4. Although there is no direct functional proof, we propose that the water elimination routes were sufficiently up-regulated to facilitate water removal. Furthermore, the differences in the pattern of cellular AQP4 localization across the astrocyte membrane, i.e., no longer solely restricted to compartments facing blood vessels, suggests a different role, probably in the formation of a glial scar, which is prominent at this phase. Indeed, pan-astrocytic AQP4 expression has been shown to enhance astrocyte migration *in vitro *and *in vivo *[31,32]. During this phase, closure of the BBB was observed which is associated with disappearance of serum protein that can be extravasated via the plasma membrane of endothelial cells back to the blood. Other potential clearing mechanisms include the digestion of serum proteins in the extracellular space by astrocytes [1,7]. Therefore, AQP4 may highly facilitate the efflux of water into the blood or the CSF along the osmotic gradient but may also facilitate astrocyte scarring and the associated uptake of the protein component of fluid. These results are consistent with previous data showing that high AQP4 expression is associated with glial scars [33,34], although the associated water content was not directly measured in these studies. Alternatively, high AQP4 expression during this phase could also be involved in regulating the fluid in newly formed cavitation due to necrosis. Nevertheless, such a mechanism is likely secondary, as AQP4 was significantly upregulated before significant cavitation appeared (no sooner than 20 dpi).

## Conclusion

In conclusion, in addition to BBB permeability, we add to the understanding the time lag in AQP4 upregulation as an additional mechanism for the exacerbation of interstitial edema. Future efforts to increase AQP4 expression by therapeutic intervention could help to prevent the deleterious occurrence of edema exacerbation.

## List of Abbreviations Used

ADC: Apparent diffusion coefficient; AQP4: Aquaporin 4; BBB: Blood brain barrier; Dpi: Day(s) post injection; DWI: Diffusion weighted images; GFAP: Glial fibrillary acidic protein; IL1β: Interleukin 1β; LPC: L-α-lysophosphatidylcholine; MRI: Magnetic resonance imaging; ROI: Region of interest; T2WI: T2 weighted images.

## Competing interests

The authors declare that they have no competing interests.

## Authors' contributions

TT participated in the study design, carried out animal experiments, participated in MR scanning, analyzed the results and drafted the manuscript. NM participated in the animal experiments and revised the manuscript. ID established and performed the MR imaging. NC established and carried out the RT-qPCR experiments. CB instructed the animal experiments and revised the manuscript. JA carried out the histological staining and western blot experiments. BB participated in the study design and revised the manuscript. CM supervised the MR imaging and revised the manuscript. KGP participated in the study design, supervised the experiments, contributed to the data interpretation and revised the manuscript. VD initiated the project, supervised the experiments, contributed to the data interpretation and revised the manuscript. All authors read and approved the final manuscript.
